# Response to *Optic nerve sheath diameter in critically ill patients: nuances and interpretation*

**DOI:** 10.1186/s13054-020-03149-1

**Published:** 2020-07-14

**Authors:** Ziyue Yang, Shuguang Zhang, Tongwen Sun

**Affiliations:** General ICU, The First Affiliated Hospital of Zhengzhou University, Henan Key Laboratory of Critical Care Medicine, Zhengzhou Key Laboratory of Sepsis, Zhengzhou, 450052 China

To the Editor:

We thank Dr. Lal and his colleagues for their attention to our study in *Critical Care*. First of all, we are very pleased to see their recommendations and acknowledge that there is still a lot of work to be done to explore the influencing factors of intracranial pressure (ICP)/optic nerve sheath diameter (ONSD). In addition to the serum albumin, serum sodium, and bedside angle mentioned in this study, there are many external factors that affect the ICP/ONSD judgment of sepsis-associated encephalopathy (SAE), including blood glucose, sedative use, albumin use, diuretic use, respiratory alkalosis, inspiration to expiration ratio, steroid use and withdrawal, and blood pressure; vasoactive drug use may affect intracranial blood flow and ICP/ONSD [1–3].

We understand the concern of Lal et al. that basic data such as hypertension and steroid history have an uncertain impact on the basal level of ICP/ONSD. The reason why it is not included in the table is that, on the one hand, most of the patients in the three groups in our study are overlapping (Fig. 1), so the medical history of patients in different groups is similar. On the other hand, the sample size of our initial study was very small, and the frequency of patients taking long-term steroids was very low in our study population.

**Fig. 1:**
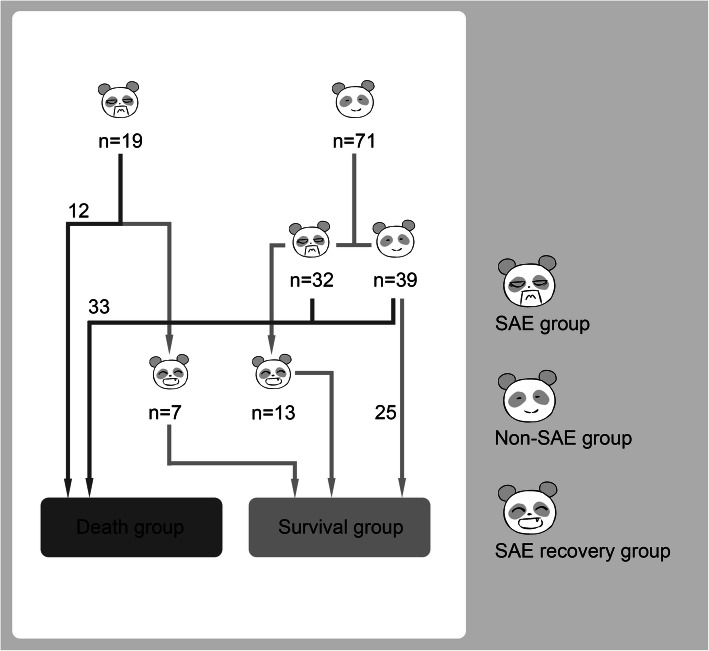
The flow chart of this study

In addition, with regard to the baseline data of blood pressure when measuring ONSD, since our small observational study measured patients when their vital signs were stable, most patients’ blood pressure was in the normal range. However, the concern is that some patients use vasopressor or antihypertensive drugs, and we are not sure whether the use of drugs will affect the measurement. Furthermore, we have summarized the blood pressure of the three groups when measuring ONSD in our previous study [4]. There was no statistical difference among the three groups.

Finally, the effects of steroid use and sudden withdrawal on ICP are not clear [5], and steroid deficiency caused by sepsis cannot be accurately quantitatively evaluated. We believe that more studies will be carried out in the future to evaluate the influencing factors of ICP/ONSD, and more studies should focus on the accuracy of ICP estimation by ONSD.

## Data Availability

All data generated or analyzed during this study are included in this published article and its supplementary information files.
